# Circulation of COVID-19-Related Medicines on Japanese Websites during the COVID-19 Pandemic and Their Quality and Authenticity

**DOI:** 10.4269/ajtmh.23-0710

**Published:** 2024-09-17

**Authors:** Shu Zhu, Naoko Yoshida, Ryo Matsushita, Mohammad Sofiqur Rahman, Kazuko Kimura

**Affiliations:** ^1^ Medi-Quality Security Institute, Graduate School of Medical Sciences, Kanazawa University, Kanazawa, Japan;; ^2^ Medicine Security Workshop, 4F Venture Business Laboratory, Kanazawa University, Kanazawa, Japan;; ^3^ AI Hospital/Macro Signal Dynamics Research and Development Center, Institute of Medical, Pharmaceutical and Health Sciences, Kanazawa University, Kanazawa, Japan;; ^4^ Clinical Pharmacy and Healthcare Sciences, Faculty of Pharmacy, Institute of Medical, Pharmaceutical and Health Sciences, Kanazawa University, Kanazawa, Japan

## Abstract

Substandard and falsified medical products for treating COVID-19 have spread worldwide. These medicines have entered Japan through personal importation of products purchased via the Internet. In this study, we investigated the circulation of 19 COVID-19-related medicines on the Internet in Japan and evaluated the pharmaceutical quality and authenticity of 2 medicines (dexamethasone tablets and ivermectin tablets) obtained online. We purchased 23 samples of 0.5-mg dexamethasone tablets and 13 samples of 3-mg ivermectin tablets from the Internet in December 2020 and July 2022. We investigated the quality and authenticity of the obtained samples through visual observation and tested their authenticity. We conducted pharmacopoeia compliance testing (quantitative assay, content uniformity tests, and dissolution tests) using the high-performance liquid chromatography–photodiode array detector method. No prescription was ever required at the time of purchase. Visual observation revealed that most samples lacked a package insert and some samples had packaging deficiencies. In terms of authenticity, eight ivermectin samples were genuine; the authenticity of the other samples remained uncertain. Four dexamethasone samples and three ivermectin samples failed quality testing based on pharmacopeia validation standards. Our findings illustrate that dexamethasone and ivermectin tablets of poor quality are available online. It is important to increase consumer awareness and provide information about these medicines to prevent the purchase of substandard medicines via the Internet.

## INTRODUCTION

The pandemic resulting from the novel coronavirus severe acute respiratory syndrome coronavirus 2 (SARS-CoV-2) has had a huge impact worldwide.^1^^,^^2^ With the public health crisis caused by the pandemic, health care systems and regulatory agencies in various countries have faced unprecedented pressure and challenges. Such threats often create an opportunity for the proliferation of substandard and falsified medical products.^3^ The increase in falsified and substandard medicines owing to the COVID-19 pandemic cannot be ignored.^4^^–^^8^ The WHO definition of “substandard and falsified medical products” is as follows:

- substandard (also called “out of specification”): these are authorized medical products that fail to meet either their quality standards or specifications, or both;
- unregistered/unlicensed: medical products that have not undergone evaluation and/or approval by the National or Regional Regulatory Authority for the market in which they are marketed/distributed or used, subject to permitted conditions under national or regional regulation and legislation;
- falsified: medical products that deliberately/fraudulently misrepresent their identity, composition, or source.^9^

Falsified versions of masks, personal protective equipment, disinfectants, and testing kits were reported in the early days of the pandemic.^7^^,^^10^^–^^12^ Subsequently, the efficacy of chloroquine and hydroxychloroquine in treating COVID-19 received widespread political and media attention, and falsified products were soon discovered in Africa.^13^^–^^15^ A falsified version of remdesivir, a medicine used to treat COVID-19, was found in Guatemala, India, and Mexico.^16^^,^^17^ Since their introduction, falsified COVID-19 vaccines have been detected in several countries worldwide.^18^^–^^20^ Even today, numerous public agencies continue to issue reports and warnings regarding falsified COVID-19 treatments and related products marketed to the public.^21^^,^^22^

The criminal activity of drug falsification became especially pervasive during the COVID-19 pandemic,^22^^,^^23^ as consumer preferences turned to purchasing goods over the Internet because of recommended measures in Japan to prevent infectious diseases that were intended to reduce physical contact among people.^24^ “Personal import” refers to the direct purchase of overseas products for personal use.^25^ In fact, the Japanese government has performed well in terms of governance. There are practically no falsified or substandard medicines in the formal distribution channels of Japan. Previous reports indicate that falsified and substandard medicines enter Japan via the personal importation of products purchased on the Internet.^26^^–^^31^

Dexamethasone has been demonstrated to improve the survival rate in patients with serious illness owing to COVID-19 and is one of the medicines recognized by the Ministry of Health, Labor and Welfare in Japan for the treatment of COVID-19.^32^^,^^33^ The therapeutic effect of ivermectin for COVID-19 is controversial,^34^ but widespread misinformation disseminated online claiming that ivermectin has preventive and therapeutic effects against COVID-19 increased general consumer awareness regarding the drug.

The escalating pandemic in Japan during 2021 resulted in a shortage of the dexamethasone supply in September of the same year.^35^ Twenty-one records of falsified dexamethasone worldwide have been filed in the WHO Global Surveillance and Monitoring System database.^36^ Substandard and falsified reports of ivermectin are also continually emerging.^37^^–^^39^ These facts suggest that Japanese consumers may be obtaining falsified dexamethasone and ivermectin through personal importation via the Internet.

In summary, there is no information on the circulation of COVID-19-related medicines on Japanese websites or their quality and authenticity. To investigate the circulation, quality, and authenticity of COVID-19 medications available via the Internet, we investigated COVID-19 medications sold online, and we purchased dexamethasone and ivermectin tablets from the Internet to conduct quality assessment and investigate the authenticity of these medicines.

## MATERIALS AND METHODS

### Reporting system and ethical approval.

This project was a shared study on the current situation and countermeasures regarding international circulation of falsified medicines conducted by the Ministry of Health, Labor and Welfare of Japan. All methods in this study were carried out in accordance with relevant guidelines and standards. The study was conducted and reported according to the Strengthening the Reporting of Observational Studies in Epidemiology (STROBE) guidelines and Medicine Quality Assessment Reporting Guidelines (MEDQUARG).^40^^,^^41^ Completed guideline checklists are included in Supplemental Tables 1 and 2. This study did not require ethical review board approval because it did not involve any animal experimentation and/or human subject research.^42^ Because relevant approval and consent have not been obtained, the names and addresses of websites and the names of manufacturers, commercial brands, and product details have been retained.

### Study settings and design.

In this study, we prospectively investigated the circulation of COVID-19-related medicines on the Internet in Japan and conducted a trial purchase survey for two COVID-19-related medicines circulating on the Internet in the form of a cross-sectional study. A mystery buyer searched each website before purchasing and purchased all available samples online within 1–2 days. The sampling method was a complete count survey.

### COVID-19-related medicines on the Internet.

We investigated 19 medicines listed as COVID-19-related medicines: dexamethasone, remdesivir, favipiravir, lopinavir plus ritonavir, nelfinavir, cepharanthine, hydroxychloroquine, chloroquine, ivermectin, ciclesonide, tocilizumab, nafamostat mesilate, casirivimab/imdevimab (genetic recombination), baricitinib, molnupiravir, sotrovimab, nirmatrelvir/ritonavir, tixagevimab/cilgavimab, and ensitrelvir.

Google is the most popular search engine in Japan.^43^ Using the search engine Google Japan, we identified websites selling these COVID-19-related medicines with the keyword search terms “(ingredient name) personal importation,” OR “(product name) personal importation” in Japanese. All sites that were identified using this search formula were surveyed between November 24, 2020 and March 1, 2022, with four of these surveys conducted at different times during this period. All data were collected manually. During the statistical analysis, the number of merchant websites and number of products (total quantity) that can be ordered from all merchant websites were manually totaled. The number of websites identified in each search varied with each medicine, which was not based on any criteria. All data were searched and counted manually by the same researcher. To ensure validity, the search results for each medicine were obtained on the same day.

### Sample collection.

We conducted nonrandom selection because, according to our investigation, dexamethasone is the most-sold COVID-19-related medicine on the Internet, followed by ivermectin. We used Google Japan to identify websites selling the target medicines (dexamethasone and ivermectin) by using the search terms “dexamethasone personal import” and “ivermectin personal import” in Japanese. All merchant websites that could be identified using Google Japan were targeted. We did not verify this based on any criteria. Regarding the selection of merchant websites for dexamethasone and ivermectin, we simulated consumer Internet searches for personal importation using search engines and purchased samples from the resultant websites. We purchased all samples from target websites where we could complete a purchase. Because we purchased all samples that can be purchased online, there is no basis for calculation. We performed Internet searches and purchased dexamethasone products between December 14, 2020 and January 15, 2021 and did the same for ivermectin products between July 1, 2022 and August 23, 2022.

We selected 0.5-mg dexamethasone tablets and 3-mg ivermectin tablets as the research targets in this study, given that these strengths were sold on all websites (Table 1).

**Table 1:** Circulation of COVID-19-related medicines on the Internet

No.	Ingredient Name (Generic Name)	Approval Date for COVID-19 in Japan (Month.Day.Year)	No. of Merchant Sites/Total No. That Can Be Ordered from All Sites on Indicated Search Date (Month.Day.Year)				
			8.27.2020	11.24.2020	3.1.2021	9.7.2021	3.1.2022
1	Dexamethasone	7.17.2020	16/18	21/22	23/24	26/41	35/48
2	Remdesivir	5.7.2020	0/0	0/0	0/0	0/0	1/1
3	Favipiravir	Unapproved	3/3	1/2	2/2	2/3	10/11
4	Lopinavir + ritonavir	Unapproved	12/17	8/13	11/17	13/20	13/22
5	Nelfinavir	Unapproved	2/2	2/2	2/2	2/6	2/6
6	Cepharanthine	Unapproved	3/3	4/4	4/4	5/5	6/6
7	Hydroxychloroquine	Unapproved	22/23	20/21	20/21	26/27	24/25
8	Chloroquine	Unapproved	4/4	3/3	3/3	4/5	4/5
9	Ivermectin	Unapproved	15/15	20/20	19/20	29/31	28/45
10	Ciclesonide	Unapproved	15/19	15/19	15/19	16/20	21/25
11	Tocilizumab	1.21.2022	1/1	1/1	1/1	1/4	1/4
12	Nafamostat mesilate	Unapproved	1/1	0/0	1/1	1/2	1/2
13	Casirivimab/imdevimab (genetic recombination)	7.19.2021	–	–	–	0/0	0/0
14	Baricitinib	4.23.2021	–	–	–	4/6	5/7
15	Molnupiravir	12.24.2021	–	–	–	0/0	8/11
16	Sotrovimab	9.27.2021	–	–	–	0/0	0/0
17	Nirmatrelvir/ritonavir	2.10.2022	–	–	–	0/0	0/0
18	Tixagevimab/cilgavimab	8.30.2022	–	–	–	0/0	0/0
19	Ensitrelvir	11.22.2022	–	–	–	0/0	0/0

– = the medicine was not within the scope of the investigation at that time point.

### Visual observation.

The purchased products were evaluated using the Tool for Visual Inspection of Medicines by the International Pharmaceutical Federation,^44^ which is an official and reliable tool.

### Purchase price.

We calculated the purchase price, excluding shipping and importation fees, for each dexamethasone and ivermectin tablet by using information available on the Internet.

### Investigation of authenticity and electronic package inserts.

On March 18, 2021 and September 13, 2022, a questionnaire that included photographs of the purchased products was sent to each manufacturer or distributor to inquire about the authenticity of their products. The questionnaire included our observations regarding appearance, the results of authenticity testing, a description of the samples (e.g., product name, active pharmaceutical ingredient [API], strength, form, manufacturer name and address, batch/lot number, manufacturing license number, logo, manufacture date, and product expiration date) as well as photographs of the outer packaging and tablets. We further asked whether 1) the product was allowed to be manufactured or sold in the manufacturing country or in Japan and 2) any countermeasures had been taken against falsified products. Because most samples lacked a package insert, on May 26, 2022 and June 28, 2023, we e-mailed manufacturers to determine whether a package insert was available electronically.

### Details provided on websites.

We observed and recorded whether websites provided the following details, as required by the Act on Specified Commercial Transactions^45^ for personal importation into Japan: name of a representative or responsible person; name, address, and telephone number of the business; product price; shipping fee; date and time of payment; date and time of product delivery; payment method; and conditions of return.

On each website, we also observed and recorded whether websites provided the following details related to the Act on Securing Quality, Efficacy and Safety of Products Including Pharmaceuticals and Medical Devices (translation of the Japanese law)^46^: a statement recommending consultation with a doctor or pharmacist, a description of personal importation, a statement outlining any purchase quantity limit, a description of the medicine, and the contact for medical consultation.

### Materials.

Dexamethasone (Tokyo Chemical Industry Co., Ltd., Tokyo, Japan), ivermectin (FUJIFILM Wako Pure Chemical Corporation, Osaka, Japan), sodium dodecyl sulfate, sodium phosphate monobasic monohydrate, 5N hydrochloric acid (Nacalai Tesque, Inc., Kyoto, Japan), acetonitrile (FUJIFILM Wako Pure Chemical Corporation), and methanol (FUJIFILM Wako Pure Chemical Corporation) were used in the analysis. All other chemicals were commercially available and of analytic grade.

### Method of pharmacopeia compliance testing.

The Japanese Pharmacopoeia 17th Edition does not publish the analysis method for ivermectin and does not classify impurities of dexamethasone. Therefore, the analytical method used in this study was based on the United States Pharmacopeia (USP) 2018 and the British Pharmacopoeia (BP) 2019.^47^^,^^48^ The analytical method was validated according to International Conference on Harmonisation guidelines.^49^ The specific validation parameters are listed in Supplemental Tables 3 and 4. Each sample was labeled with a sample code regardless of packaging.

### Pharmacopeia testing of tablets.

Before analyzing the samples, we analyzed original dexamethasone (trade name: Decadron® 0.5-mg tablet) and ivermectin (trade name: Stromectol® 3-mg tablet) products that are regularly distributed in Japan (officially purchased for research purposes). We tested all dexamethasone samples and 12 of 13 ivermectin samples. One of the ivermectin samples (sample no. 7) was not tested because the number of tablets was insufficient.

### Quantitative assay.

#### Dexamethasone.

We used the LC-10 AD system (Shimadzu, Kyoto, Japan) supported by a photodiode array detector for the quantitative assay, which was based on the BP 2019.^47^ Using a Shim-pack CLC-ODS column (particle size, 5 µm; 25 cm × 4.6 mm inside diameter; Shimadzu), we maintained the column temperature at 45°C using a column oven (CTO-20AC; Shimadzu). We used a mobile phase A of acetonitrile-containing water (1:3, vol/vol) and a mobile phase B of acetonitrile-containing water (3:7, vol/vol) with the following elution gradients: A/B = 100/0 (0–15 minutes), 0/100 (15–40 minutes), and 100/0 (40–45 minutes). The mobile phase flow rate was 1.2 mL/min, the injection volume was 20 µL, and the detection wavelength was 254 nm. Measurements for dexamethasone were performed using 10 tablets for each sample. The API content relative to the labeled amount was determined. In accordance with the BP 2019, the average passing API content rate in 10 tablets was determined to be 97–103%^47^; samples that did not reach this criterion were assayed in the second stage. Components were identified by confirming that the retention time of the component matched that of the standard reagent and that their ultraviolet (UV) spectra coincided.

#### Ivermectin.

For analysis of ivermectin, the high-performance liquid chromatography (HPLC) system comprised a system controller ORGANIZER, HPLC pump L-2130, autosampler L-2200, intelligent column oven L-2300, a 1430 diode array detector, and a ChromAssist chromatography data station (Hitachi High-Technologies Corporation, Tokyo, Japan). The UV detection was at 245 nm, and the column oven was set at 30°C. The analytical column used was a Gemini-NX 5-µm C18 110-Å P/N 00F-4454-E0 (Phenomenex, Torrance, CA), the injection volume was 20 µL, and the flow rate was 1.2 mL/min. The mobile phase consisted of acetonitrile, methanol, and water (53:35:12, vol/vol/vol). The API content relative to the labeled amount was determined. In accordance with the USP 2018, the average passing API content rate in 10 tablets was determined to be 90–110%.^48^ If a sample did not reach this criterion, it was assayed in the second stage. Components were identified by confirming that the retention time of the component matched that of the standard reagent and that their UV spectra coincided.

### Content uniformity testing.

Content uniformity testing of dexamethasone and ivermectin was based on the BP 2019 and the USP 2018.^47^^,^^48^ In the first stage, a sample acceptance value of ≤15 was determined to be a passing value for tablets^47^^,^^48^; samples that did not reach this criterion were assayed in the second stage.

### Assay of dexamethasone for specified impurities.

An assay of dexamethasone for specified impurities was conducted in accordance with the BP 2019.^47^ We tested 5 types of specified impurity (B, G, F, J, and K) among 11 types of impurity (A–K) in this study. The BP 2019 indicates that specified impurities B, F, J, and K shall not exceed the area of the principal peak by more than 3 times, and specified impurity G shall not exceed this area by more than 1.5 times.^47^

### Dissolution testing.

Dissolution testing was performed using the NTR-VS6P dissolution apparatus (Toyama Sangyo Co. Ltd., Osaka, Japan) in accordance with the USP 2018.^48^ Six tablets per sample were measured. A tolerance of ≥75% of the labeled amount of dexamethasone and a tolerance of ≥85% of the labeled amount of ivermectin at a dissolution time of 45 minutes were judged as passing the dissolution test in the first stage.^48^ Samples that did not reach these tolerance levels were assayed in the second stage.

## STATISTICAL ANALYSES

The means, SDs, coefficients of variation, and graphs for figures were calculated and generated using Microsoft Excel 2016.

## RESULTS

### COVID-19-related medicines on the Internet.

The results of the surveys conducted from November 2020 to March 2022 are shown in Table 1. Of the 19 medicines investigated, 14 were sold on websites for personal importation. Among them, dexamethasone was available on the largest number (22%) of websites, followed by ivermectin (19%), and the availability of these two medicines increased approximately 50% from 2020 to 2022. Therefore, we chose to investigate dexamethasone in 2020 and ivermectin in 2022 in this study. Notably, no sales information for baricitinib and molnupiravir was available before March 1, 2021^50^; sales information for these drugs appeared online immediately after they were approved in Japan.

### Sample collection.

We obtained 23 samples of 0.5-mg tablets of four brands (A, B, C, D) of dexamethasone from 18 websites and 13 samples of 3-mg tablets of four brands (E, F, G, H) of ivermectin from nine websites. Tables 2 and 3 list specific information for the samples collected. No prescription was required by any of the websites. The shipping method for all samples was by mail, and all samples were received 1 month after purchasing. Dexamethasone samples were received between January 19, 2021 and February 4, 2021, and ivermectin samples were received between July 14, 2022 and August 29, 2022. We placed every sample in an individual zip-closure bag and securely stored the samples in an air-conditioned room (20–25°C) until analysis.

**Table 2:** List of dexamethasone samples purchased online

Sample no.	Brand	Strength (mg)	Outer Packaging	Language(s)	Labeled Manufacturing and/or Distributing Country	Shipping Country	Package Insert and Language	Authenticity	Site No.
1–13	A	0.5	Bottle	Traditional Chinese and English	Hong Kong	Taiwan	None	Unknown	1–13
14–17	B	0.5	Bottle	Traditional Chinese and English	Hong Kong	Taiwan	None	Unknown	4, 6, 11, 13
18–20	C	0.5	Box	English	India	Hong Kong	None	Unknown	13–15
21, 22	C	0.5	Box	English	India	Hong Kong	None	Unknown	16, 17
23	D	0.5	Plastic bag	English	India	India	None	Unknown	18

**Table 3:** List of ivermectin samples purchased online

Sample No.	Brand	Strength (mg)	Outer Packaging	Language(s)	Labeled Manufacturing and/or Distributing Country	Shipping Country	Package Insert /Language	Authenticity	Site No.
1	E	3	Box	English and French	Netherlands	Taiwan	Yes/English and French	Genuine	1
2, 3	E	3	Box	English and French	Netherlands	Taiwan	Yes/English and French	Genuine	6, 8
4	E	3	Box	English and French	Netherlands	Hong Kong	Yes/English and French	Genuine	5
5	E	3	Box	English and French	Netherlands	Taiwan	Yes/English and French	Genuine	3
6	E	3	Box	English and French	Netherlands	Taiwan	Yes/English and French	Genuine	2
7	E	3	Box	English and French	Netherlands	Taiwan	Yes/English and French	Genuine	3
8	F	3	Box	English	India	India	None	Unknown	4
9	F	3	Box	English	India	Taiwan	None	Unknown	9
10	F	3	Clear plastic case	English	India	India	None	Unknown	7
11, 12	G	3	Box	English	France	Taiwan	Yes/English	Unknown	6, 8
13	H	3	Box	English	India	India	Yes/English	Unknown	4

### Visual observation.

#### Dexamethasone samples.

The purchased products included 23 samples of four brands in three packaging forms. Seventeen samples were of two brands (A, B) with bottle-type packaging and no obvious problems with the product information found. Five samples were of another brand (C) with box-type packaging; however, all boxes showed signs of having been opened, and some samples lacked any information on the package or were marked with a ballpoint pen. One sample was the fourth brand (D) and was packaged in a transparent plastic zip-closure bag (blisters were inside the plastic bag); the sample lacked original outer packaging or product information and did not match the website picture, which was of a boxed product. Stains were observed on the paper backing of blisters among samples that were not bottled (C and D products); however, no stains were found on the tablets themselves after the outer packaging was removed. All samples lacked any instructions. We also did not find any “quick response” (QR) codes or barcodes for an electronic package insert.

#### Ivermectin samples.

Among the 13 samples purchased, 7 were original ivermectin products (no. 1–7; brand E) with no obvious problems found; the packaging label stated that the product was manufactured in the Netherlands. Among three samples of generic ivermectin (no. 8–10; brand F), one with clear plastic case-type packaging lacked information and did not match the website picture, which was of a boxed product. The three samples lacked any instructions. We also did not find any QR codes or barcodes for an electronic package insert. The packaging was labeled as being manufactured in India. For two samples (no. 11 and 12; brand G) of generic ivermectin with no obvious problems, the packaging label stated that the product was manufactured in France. The last sample was generic ivermectin (no. 13; brand H) with no obvious problems found; the packaging label stated that the product was manufactured in India.

### Purchase price.

**Figure 1.**
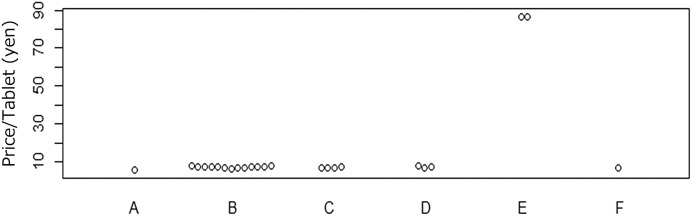
Price per tablet (shipping fee excluded) for 0.5-mg dexamethasone tablets purchased in Japan and online. (A) Branded product in Japan (official price); (B) brand A product online (*N* = 13); (C) brand B product online (*N* = 4); (D) brand C product online (*N* = 3); (E) brand C product online (*N* = 2); and (F) brand D product online (*N* = 1).

**Figure 2.**
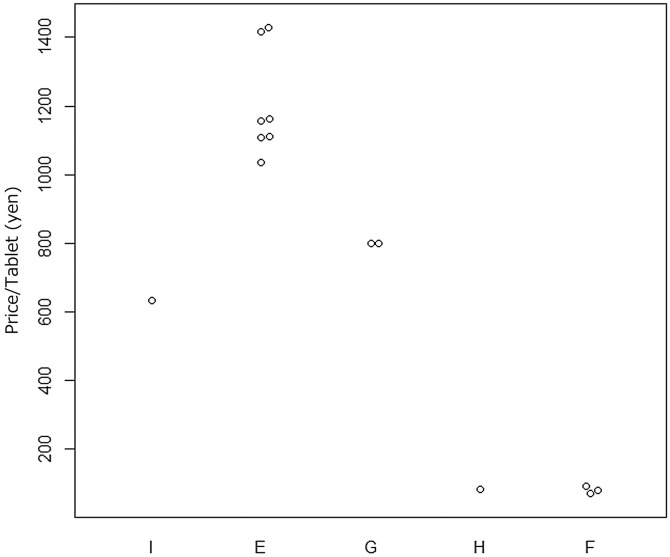
Price per tablet (shipping fee excluded) for 3-mg ivermectin tablets purchased in Japan and online. (I) Branded product in Japan (official price); (E) brand E product online (*N* = 7); (G) brand G product online (*N* = 2); (H) brand H product online (*N* = 1); and (F) brand F product online (*N* = 3).

Dexamethasone and ivermectin are covered by the National Health Insurance medicine price list in Japan. However, these medicines are covered by the National Health Insurance and therefore have official prices in Japan. The official prices of dexamethasone 0.5-mg tablets and ivermectin 3-mg tablets were 5.7 JPY/tablet and 632.9 JPY/tablet, respectively, for the branded products at the time of our purchase of online samples. Additionally, medical insurance covers 30% of the cost of medicine; 30% of the medicine price is paid by the patient. However, personally imported medicines are not subject to these regulations. Personally imported medicines may be higher or lower than the official price in Japan. We calculated the average price of each product and listed the average prices with the official prices in Japan in Figures for comparison. The average prices for one tablet of dexamethasone 0.5 mg and one tablet of ivermectin 3 mg purchased on the Internet were higher than the prices of these tablets sold at pharmacies in Japan (Figures 1 and 2). However, it is worth noting that ivermectin samples of brands F and H had low prices, with an average of 98 ± 20 JPY/tablet and 80 JPY/tablet, respectively.

### Authenticity and electronic package inserts.

For dexamethasone, one manufacturer of brand B responded to our survey but did not address the authenticity of the product. At the time of this writing, we have not received any response from the other three manufacturers. Thus, the authenticity of all 23 dexamethasone samples remains unknown. For ivermectin, the manufacturer of the original ivermectin product Stromectol^®^ (MSD K.K.) responded that for product E, “all 8 samples are genuine.” The identification method regarding the authenticity of medicines is determined according to each company’s internal regulations. MSD K.K. stated that the authenticity identification and analysis methods could not be disclosed because these comprised internal information of the company. We did not receive any response from the other three manufacturers. The authenticity of the other five ivermectin samples remains unknown.

Similarly, we did not receive a response from the manufacturers of dexamethasone and ivermectin samples that did not include a package insert regarding the availability of an electronic version of the package insert.

### Evaluation of website details.

We bought all purchasable samples from identified searchable websites for this study. Among dexamethasone samples purchased from 18 websites and ivermectin samples purchased from 9 websites, 5 and 6 websites, respectively, advertised and sold their products as treatment of COVID-19, including websites in which customer reviews mentioned use for COVID-19. The language of all 27 websites was Japanese. Tables 4–7 show the results of items on the identified websites related to the Specified Commercial Transactions Law and the Act on Securing the Efficacy and Safety of Products Including Pharmaceuticals and Medical Devices.

**Table 4:** Websites selling dexamethasone according to requirements of the Specified Commercial Transactions Law

Required Details	No. of Websites (%)*
Name of a representative or responsible person	13 (72.0)
Name of business	17 (94.0)
Address of business	18 (100.0)
Telephone number	15 (83.0)
Product price	18 (100.0)
Shipping fee	18 (100.0)
Payment date and time	16 (89.0)
Product delivery date and time	18 (100.0)
Payment method	18 (100.0)
Conditions for return	18 (100.0)

* *N* = 18 websites.

**Table 5:** Websites selling dexamethasone according to the Act on Securing Quality, Efficacy and Safety of Products Including Pharmaceuticals and Medical Devices

Required Details	No. of Websites (%)*
Statement recommending consultation with a doctor or pharmacist	14 (78.0)
Description of personal importation	16 (89.0)
Statement on the purchase quantity limit	16 (89.0)
Description of unapproved medicines or ethical medicines	18 (100.0)
Name of the product	18 (100.0)
Photograph of the product	18 (100.0)
Concentration of the active ingredient	11 (61.0)
Description of the active pharmaceutical ingredient	12 (67.0)
Description of potential side effects	11 (61.0)

* *N* = 18 websites.

**Table 6:** Websites selling ivermectin according to requirements of the Specified Commercial Transactions Law

Required Details	No. of Websites (%)*
Name of a representative or responsible person	8 (89.0)
Name of business	8 (89.0)
Address of business	8 (89.0)
Telephone number	9 (100.0)
Product price	9 (100.0)
Shipping fee	9 (100.0)
Payment date and time	9 (100.0)
Product delivery date and time	9 (100.0)
Payment method	9 (100.0)
Conditions for return	9 (100.0)

* *N* = 9 websites.

**Table 7:** Websites selling ivermectin according to the Act on Securing Quality, Efficacy and Safety of Products Including Pharmaceuticals and Medical Devices

Required Details	No. of Websites (%)*
Statement recommending consultation with a doctor or pharmacist	8 (89.0)
Description of personal importation	9 (100.0)
Statement on the purchase quantity limit	9 (100.0)
Description of unapproved medicines or ethical medicines	
Name of the product	9 (100.0)
Photograph of the product	8 (89.0)
Concentration of the active ingredient	8 (89.0)
Description of the active pharmaceutical ingredient	8 (89.0)
Description of potential side effects	8 (89.0)

* *N* = 9 websites.

### Pharmacopeia compliance testing of tablets.

The results of the original dexamethasone (trade name: Decadron tablets) and ivermectin (trade name: Stromectol 3 mg tablet) products showed that they met USP standards and met the API content criteria. Detailed results are presented in Supplemental Tables 5 and 6.

### Dexamethasone.

Table 8 presents the results of pharmacopeia compliance testing of dexamethasone. In quantitative analysis, 19 samples (82.6%) passed and 4 samples (17.4%) failed. Twenty-three samples (100%) passed content uniformity testing and dissolution testing. Specified impurities were not detected in any of the samples tested in this study.

**Table 8:** Results of quantity, content uniformity, and dissolution testing of dexamethasone tablets

Sample No.	Strength (mg)	Quantitative Analysis		Content Uniformity Test		Dissolution Test		Any Fail
		Mean Content (%) ± SD	Judgment	Acceptance Value	Judgment	Mean Dissolution Rate (%) ± SD	Judgment	Judgment
1	0.5	98.1 ± 0.7	Pass	2.2	Pass	99.4 ± 5.6	Pass	Pass
2	0.5	98.8 ± 2.0	Pass	4.9	Pass	96.5 ± 4.5	Pass	Pass
3	0.5	103.4 ± 2.2	Fail	7.1	Pass	102.5 ± 3.4	Pass	Fail
4	0.5	102.3 ± 1.1	Pass	3.3	Pass	96.8 ± 1.9	Pass	Pass
5	0.5	99.5 ± 2.7	Pass	6.5	Pass	96.3 ± 1.4	Pass	Pass
6	0.5	97.4 ± 0.9	Pass	3.4	Pass	82.6 ± 3.6	Pass	Pass
7	0.5	98.4 ± 2.4	Pass	6.0	Pass	88.8 ± 16.1	Pass	Pass
8	0.5	100.2 ± 1.0	Pass	2.5	Pass	96.9 ± 1.9	Pass	Pass
9	0.5	98.5 ± 1.0	Pass	2.5	Pass	97.0 ± 1.1	Pass	Pass
10	0.5	98.7 ± 1.6	Pass	3.8	Pass	99.3 ± 3.4	Pass	Pass
11	0.5	98.4 ± 2.4	Pass	6.0	Pass	98.7 ± 2.4	Pass	Pass
12	0.5	97.5 ± 1.0	Pass	1.4	Pass	101.4 ± 9.4	Pass	Pass
13	0.5	97.0 ± 1.9	Pass	6.0	Pass	86.7 ± 4.1	Pass	Pass
14	0.5	101.0 ± 3.7	Pass	8.8	Pass	96.1 ± 2.4	Pass	Pass
15	0.5	102.3 ± 6.2	Pass	13.2	Pass	98.4 ± 2.0	Pass	Pass
16	0.5	98.3 ± 3.2	Pass	7.8	Pass	98.0 ± 3.8	Pass	Pass
17	0.5	101.3 ± 1.5	Pass	3.5	Pass	101.5 ± 3.6	Pass	Pass
18	0.5	103.4 ± 2.6	Fail	8.2	Pass	97.0 ± 1.0	Pass	Fail
19	0.5	101.6 ± 4.7	Pass	11.4	Pass	89.4 ± 1.6	Pass	Pass
20	0.5	105.4 ± 3.4	Fail	11.9	Pass	91.4 ± 5.1	Pass	Fail
21	0.5	103.7 ± 8.2	Fail	14.6	Pass	89.3 ± 2.7	Pass	Fail
22	0.5	101.5 ± 3.8	Pass	9.1	Pass	89.8 ± 2.0	Pass	Pass
23	0.5	97.3 ± 1.5	Pass	4.9	Pass	88.8 ± 1.9	Pass	Pass

### Ivermectin.

Table 9 presents the results of pharmacopeia compliance testing of ivermectin. Ten tested samples (83.3%) passed and two samples (16.7%) failed in quantitative analysis. Twelve tested samples (100%) passed content uniformity testing. Nine tested samples (70.0%) passed and three samples (30.0%) failed dissolution testing.

**Table 9:** Results of quantity, content uniformity, and dissolution testing of ivermectin tablets

Sample No.	Strength (mg)	Quantitative Analysis		Content Uniformity Test		Dissolution Test		Any Fail
		Mean Content (%) ± SD	Judgment	Acceptance Value	Judgment	Mean Dissolution Rate (%) ± SD	Judgment	Judgment
1	3	98.6 ± 2.0	Pass	4.8	Pass	105.5 ± 3.4	Pass	Pass
2	3	102.9 ± 2.4	Pass	5.7	Pass	85.6 ± 0.8	Pass	Pass
3	3	98.3 ± 3.9	Pass	9.6	Pass	101.9 ± 3.3	Pass	Pass
4	3	104.6 ± 1.1	Pass	2.6	Pass	94.8 ± 3.4	Pass	Pass
5	3	102.1 ± 1.8	Pass	4.3	Pass	97.8 ± 2.3	Pass	Pass
6	3	106.0 ± 3.8	Pass	9.2	Pass	106.1 ± 3.6	Pass	Pass
7	3	–	–	–	–	–	–	–
8	3	106.6 ± 4.2	Pass	10.1	Pass	76.0 ± 24.4	Fail	Fail
9	3	80.7 ± 3.8	Fail	9.1	Pass	64.9 ± 13.3	Fail	Fail
10	3	83.0 ± 5.4	Fail	10.8	Pass	76.6 ± 17.1	Fail	Fail
11	3	100.1 ± 1.5	Pass	3.6	Pass	90.8 ± 5.8	Pass	Pass
12	3	101.7 ± 5.0	Pass	12.1	Pass	100.9 ± 5.0	Pass	Pass
13	3	92.7 ± 3.1	Pass	7.4	Pass	102.9 ± 4.3	Pass	Pass

– = the sample was not tested because of an insufficient number of tablets.

## DISCUSSION

In this investigation, we confirmed the presence and quality of medicines related to COVID-19 being sold in Japan via the Internet. When baricitinib and molnupiravir were approved as COVID-19 therapeutic medicines in Japan,^50^ sales information for these medicines appeared online immediately (Table 1). It can be seen from reports of the WHO and INTERPOL, as well as our survey results, that online sellers take advantage of public opinion and people’s psychological need to control and adjust the types and quantities of medicines available to them, which leads to the illegal distribution and use of medicines.^7^^,^^22^ According to our results, we speculate that consumer purchases of COVID-19-related medicines through the Internet have also been increasing. Because the quality and authenticity of these medicines have not been confirmed, consumers should avoid purchasing COVID-19-related medicines online. The quality and authenticity of COVID-19-related medicines available on the Internet need to be clarified.

For dexamethasone, multiple samples of the same product (brand A, 13 samples; 57%) were among the samples purchased. These samples were purchased from different websites, and all originated from the same shipping source, suggesting that the same supplier and inventory management agency controlled these products. Irrespective of package type, some samples did not meet the packaging standards for regular medicinal products.^51^ Such packaging has an adverse effect on the quality of the supplied medicines.^52^ Spots found on tablet packaging may have been caused by inadequate techniques used in the packaging manufacturing process or incorrect medicine management and storage. These deficiencies in packaging can lead to deleterious effects on consumer health.^52^ Furthermore, in our analysis, 18% of websites did not even mention the name of the seller, bringing into question the reliability of the website operator. However, all purchased samples lacked package inserts or other informational material, and there was no available method to obtain electronic package inserts. The necessary specific and detailed instructions for patients were lacking. In such cases, the consequence is an increased probability of the incorrect use of medicines and harmful adverse events. The results of quantitative analysis deviated only slightly from the pharmacopeia standards, and no samples displayed excessively low or high contents. The quality of the samples tested was generally good. Consumers must nevertheless avoid the purchase and improper use of these medicines from the Internet.

For ivermectin, the results for samples of the four brands (E, G, F, H) of ivermectin showed that websites for the purchase of products E and G were different from those for the purchase of products F and H (Table 3). We can infer that the circulation channels for these products were different. The most frequently purchased samples (brand E, seven samples; 54%) were genuine original products. In quality testing, all samples of brand E (*N* = 7) passed. Therefore, we can speculate that ivermectin products sold on the Internet are likely to be genuine. We purchased the second most frequently available ivermectin samples (brand F, three samples; 23%); in quality testing, all samples of brand F (*N* = 3) failed. As of this writing, we have not received any reply regarding the authenticity of product F; thus, its authenticity remains unknown. One sample of brand F lacked instructions and exhibited irregular medicine packaging, such as secondary packaging.^51^ Additionally, the sample was purchased from a website that lacked the contact address and name of a responsible person. Our previous research showed that there is a higher probability of purchasing falsified products from websites that do not give full contact details than from websites that provide these details.^28^^,^^29^ Moreover, the price of products F and H was relatively low, only one-sixth the official price of the regular medicine in Japan. In previous research, we found that the price of falsified products is generally lower than that of genuine products.^28^^–^^30^ In Australia, the existence of falsified ivermectin has been reported.^37^^,^^38^ Judging from photos in those reports, those products were the same as products F and H in our study, except that the strengths were different. Therefore, we can deduce that the brand F samples purchased online were highly likely to be falsified products with low prices. The labeled manufacturing country of brand F samples was India. Because we have not yet received a reply regarding the authenticity of product F, we cannot confirm whether the product was actually manufactured in India. The relevant regulatory authorities should pay close attention to such products and investigate whether ivermectin products manufactured in India have any potential quality problems.

Dexamethasone and ivermectin are medicines for which a prescription is required for purchase in Japan. It is extremely dangerous to take dexamethasone and ivermectin without the proper product information and guidance of a doctor. The purchase price is often one of the motivations for consumers to use online sources to purchase medicine.^53^ In terms of the online price in this study (excluding brand H and F products), we confirmed that purchasing these medicines online offers no cost advantage. However, given the shortage of medical resources during consecutive COVID-19 epidemic waves, the online purchase of medicines is considered quicker and more convenient than purchasing these in a pharmacy, encouraging the importation of medicines that can cause adverse effects if improperly used. Additionally, we believe that some individuals purposely purchased dexamethasone and ivermectin online with the expectation of a therapeutic effect against COVID-19, based on customer reviews posted on websites. Customer reviews during the COVID-19 pandemic represent an additional factor that supports the sale of dexamethasone and ivermectin on the Internet. However, online sales of prescription medicines in Japan are not allowed, even if a prescription is obtained. Medicines containing ivermectin and dexamethasone as the API have been approved and sold in Japan. However, the samples obtained in this study were not products approved in Japan, and even if obtained via Internet, these were still unofficial. According to our survey on COVID-19-related medicines available on the Internet, the number of merchant websites and the total quantity of medicines have increased over time between 2020 and 2022. Admittedly, these medicines may have been advertised on Japanese sites at some time. Previous studies have pointed out the risk that falsified and substandard medicines may be obtained through personal import via the Internet in Japan.^26^^–^^31^ Although Japan’s regulations are believed to be effective to a certain extent, loopholes remain that, if exploited, lead to extremely dangerous behavior in terms of health and hygiene. This has resulted in the need to constantly monitor the situation and take measures such as the tightening of regulations. Our research results can provide a reference for the Japanese Ministry of Health, Labor and Welfare to develop effective regulatory methods and measures in this regard. Clearly, medicines are available for personal import from the Internet, which is an inappropriate distribution route. The Ministry of Health, Labor and Welfare of Japan reminds citizens not to personally import such medicines. Strengthening of measures to prevent and control the abuse of dexamethasone and ivermectin as designated COVID-19 treatments is needed, especially during periods with more COVID-19 infections.

During a public emergency such as the COVID-19 pandemic, the demand for treatment medications will become extremely high. The global circulation of pharmaceuticals has undergone tremendous changes, and there are few cases involving the collection and analysis of relevant intelligence or data. Empirical data remain scarce on the actual prevalence, key characteristics, and economic impact of substandard and falsified medicines globally.^54^ Without reliable and robust data on substandard and falsified medicines, specifying and advocating for interventions to limit the availability of substandard and falsified medicines is difficult. Although the acute phase of the COVID-19 pandemic appears to be waning globally, our research is valuable, in that we describe the quality and circulation of medicines related to COVID-19 that are available on the Internet to a certain extent. The findings of this study can help to promote and maintain public health, and the experiences during the pandemic can serve as a reference for medicine supervision during a future public health emergency.

This study has several limitations. First, we tested only dexamethasone and ivermectin purchased online. Furthermore, we focused our research exclusively on dexamethasone and ivermectin obtained from websites marketing medicines in Japanese. As such, our results do not reflect the quality of all medicines available online. Because we collected samples of all products offered on Japanese websites during the study period, the information obtained from products offered under these conditions is unbiased and representative of the current situation at the time. However, this information is limited to products available on Japanese websites during the study period and does not represent all products currently in circulation. Additionally, the advertised products and stock status may vary depending on the time. Therefore, even if all products available during the study period were purchased, we may not have identified all dexamethasone and ivermectin products available on Japanese merchant sites. It is difficult to extrapolate the results of this study to other medicines, because manufacturers, production volumes, and distribution volumes vary. Expanding the analysis to other therapeutic products for COVID-19 would provide more comprehensive information and additional relevant findings. Second, not all manufacturers completed the authenticity survey or provided feedback regarding whether a package insert was available in an electronic format. Therefore, these aspects of the tested products could not be confirmed. Our findings suggested that active cooperation between manufacturers and regulators is needed to improve the quality of medicines available online. Third, the limited analytical conditions in this study did not allow us to detect specified impurities of dexamethasone at low concentrations. For comprehensive and accurate results regarding impurities, more precise analytical equipment and conditions may be necessary. Nevertheless, these limitations do not affect the internal and external validity and interpretation of the results.

## CONCLUSION

In this study, we found that poor-quality ivermectin was introduced into Japan via purchase on the Internet during the COVID-19 pandemic. The quality of dexamethasone purchased online was generally good, but the lack of important product information and inappropriate product management were identified problems. Internet sellers took advantage of an insufficient supply of medicines and consumers’ psychological needs during the pandemic, resulting in the inappropriate distribution and use of medicines. Consumers should be fully aware of the dangers of purchasing medicines online and should avoid purchasing medicines from the Internet. Information and warnings must be provided to encourage consumers not to purchase medicines online. The monitoring and control of illegal medicines should also be strengthened, and websites that illegally sell medicines should be promptly blocked.

## Supplemental Materials

10.4269/ajtmh.23-0710 Supplemental Table 1

10.4269/ajtmh.23-0710 Supplemental Table 2

10.4269/ajtmh.23-0710 Supplemental Table 3

10.4269/ajtmh.23-0710 Supplemental Table 4

10.4269/ajtmh.23-0710 Supplemental Table 5

10.4269/ajtmh.23-0710 Supplemental Table 6
