# Supplementary material for: Circulation of COVID-19-Related Medicines on Japanese Websites during the COVID-19 Pandemic and Their Quality and Authenticity
Source: Am J Trop Med Hyg. 2024 Sep 17;111(5):1097–106. doi: 10.4269/ajtmh.23-0710 (PMC11542516; doi:10.4269/ajtmh.23-0710)
Supplement: Supplemental Table 1 [file tpmd230710.SD1.pdf]

Supplemental Table 1. STROBE Statement—Checklist of items that should be included in reports of ***cross-sectional studies***

	**Item No**	**Recommendation**	**Line No(s)**	**Remarks**
**Title and abstract**	1	(*a*) Indicate the study's design with a commonly used term in the title or the abstract	Lines 3–4	Completed
		(*b*) Provide in the abstract an informative and balanced summary of what was done and what was found	Lines 42-60	Completed
**Introduction**				
Background/rationale	2	Explain the scientific background and rationale for the investigation being reported	Lines 62-112	Completed
Objectives	3	State specific objectives, including any prespecified hypotheses	Lines 109-112	Completed
**Methods**				
Study design	4	Present key elements of study design early in the paper	Lines 130–134	Completed
Setting	5	Describe the setting, locations, and relevant dates, including periods of recruitment, exposure, follow-up, and data collection	Lines 130–168	Completed
Participants	6	(*a*) Give the eligibility criteria, and the sources and methods of selection of participants	Lines 154-164	Completed
Variables	7	Clearly define all outcomes, exposures, predictors, potential confounders, and effect modifiers. Give diagnostic criteria, if applicable	Not Applicable	Not Applicable
Data sources/ measurement	8*	For each variable of interest, give sources of data and details of methods of assessment (measurement). Describe comparability of assessment methods if there is more than one group	Lines 212-278	Completed
Bias	9	Describe any efforts to address potential sources of bias	Lines 132-134;159-162	Completed
Study size	10	Explain how the study size was arrived at	Lines 134; 142-147;154-168	Completed

Quantitative variables	11	Explain how quantitative variables were handled in the analyses. If applicable, describe which groupings were chosen and why	Tables 8 and 9 The average value of all numbers was used.	Completed
Statistical methods	12	(*a*) Describe all statistical methods, including those used to control for confounding	Lines 147-152;280-282	Completed
		(*b*) Describe any methods used to examine subgroups and interactions	This research did not involve subgroups.	Not Applicable
		(*c*) Explain how missing data were addressed	This research did not involve missing data.	Not Applicable
		(*d*) If applicable, describe analytical methods taking account of sampling strategy	The sampling method was a complete count survey.	Not Applicable
		(*e*) Describe any sensitivity analyses	This research did not involve sensitivity analyses.	Not Applicable
**Results**				
Participants	13*	(a) Report numbers of individuals at each stage of study—eg numbers potentially eligible, examined for eligibility, confirmed eligible, included in the study, completing follow-up, and analysed	Lines 286-287;296-298	Completed
		(b) Give reasons for non-participation at each stage	This research did not involve non–participation.	Not Applicable
		(c) Consider use of a flow diagram	The sampling method was a complete count survey	Reported. The sampling method was a complete count survey
Descriptive data	14*	(a) Give characteristics of study participants (eg demographic, clinical, social) and information on exposures and potential	Lines 285–304	Completed

		confounders		
		(b) Indicate number of participants with missing data for each variable of interest	Not Applicable	Not Applicable
Outcome data	15*	Report numbers of outcome events or summary measures	Lines 286–287; 296–298	Completed
Main results	16	(*a*) Give unadjusted estimates and, if applicable, confounder-adjusted estimates and their precision (eg, 95% confidence interval). Make clear which confounders were adjusted for and why they were included(a)	Lines 285–388	Completed
		(*b*) Report category boundaries when continuous variables were categorized	Not Applicable	Not Applicable
		(*c*) If relevant, consider translating estimates of relative risk into absolute risk for a meaningful time period	Not Applicable	Not Applicable
Other analyses	17	Report other analyses done—eg analyses of subgroups and interactions, and sensitivity analyses	Not Applicable	Not Applicable
**Discussion**				
Key results	18	Summarise key results with reference to study objectives	Lines 390–477	Completed
Limitations	19	Discuss limitations of the study, taking into account sources of potential bias or imprecision. Discuss both direction and magnitude of any potential bias	Lines 490–512	Completed
Interpretation	20	Give a cautious overall interpretation of results considering objectives, limitations, multiplicity of analyses, results from similar studies, and other relevant evidence	Lines 446–489	Completed
Generalisability	21	Discuss the generalisability (external validity) of the study results	Lines 493–496	Completed
**Other information**				
Funding	22	Give the source of funding and the role of the funders for the present study and, if applicable, for the original study on which the present article is based	Lines 530–532	Completed
