# Supplementary material for: Circulation of COVID-19-Related Medicines on Japanese Websites during the COVID-19 Pandemic and Their Quality and Authenticity
Source: Am J Trop Med Hyg. 2024 Sep 17;111(5):1097–106. doi: 10.4269/ajtmh.23-0710 (PMC11542516; doi:10.4269/ajtmh.23-0710)
Supplement: Supplemental Table 2 [file tpmd230710.SD2.pdf]

Supplemental Table 2. MEDQUARG checklist

Item	Section & Topic	Description	Location in the manuscript	Remarks
1	Title/abstract/keywords	- Identify the article as a study of medicine quality (recommended MeSH headings: "medicine quality, substandard, degraded, counterfeit") - Provide an abstract of what was done and what was found, describing the main survey methods and chemical analysis techniques used	Title: lines 3–4<br>Abstract: lines 42–60<br>Keywords: lines 31–32	Completed
2	Introduction	- Summarise previous relevant drug quality information and describe the drug regulatory environment - State specific objectives	Lines 62-112	Completed
	Methods			
3	Survey details	The timing and location of the survey; when samples collected and when samples analysed.	Lines 136–168	Completed
4	Definitions	The definitions of counterfeit, substandard, and degraded medicines used.	Lines 70–77	Completed
5	Outlets	The type, including indices of size (e.g., turnover), of drug outlets sampled.	Lines 154–168	Completed
6	Sampling design	a. Sampling design and sample size calculation<br>b. Type and number of dosage units purchased/outlet<br>c. Definition of sampling frame<br>d. Question of interest, assumptions, sampling method(s) (including method of randomisation if random sampling used)	a. Lines 129–134; Lines 159–164<br>b. Lines 167–168<br>c. Lines 162–164<br>d. Lines 154–156	Completed
7	Samplers	Who carried out the sampling and in what guise? What did the collector say in buying the medicines?	Lines 132–134<br>Lines 159-162	Completed

8	Statistical method	Describe the data analysis techniques used.	Lines 147–152<br>Lines 280–282	Completed
9	Ethical issues	Whether ethical approval was sought and whether the study encountered any ethical issues.	Lines 123–124	Reported. This research did not involve ethics.
10	Packaging	Packaging examination and reference standards.	Lines 170–173	Completed
11	Chemical analysis	Chemical analysis and dissolution testing SOPs and location(s) of laboratory. Description of validation and reference standards used	Lines 204–210<br>Lines 212–219	Completed
12	Method validation	Details of laboratory method validation results, including but not limited to: Certificate of analysis for reference standard, within and between run repeatability (RSD% for n = 5–8), detection and quantitation limits, accuracy observed for reference samples, linear range for all analytes, sample preparation recovery studies, selectivity. Possibly, validation against a reference method or inter-laboratory study.	Lines 216–218	Completed
13	Blinding	Whether chemistry was performed blinded to packaging and vice versa	Lines 218–219	Completed
	Results			
14	Outlets	The details of the outlets actually sampled, "class" of pharmacy (e.g., public, private for profit, private not for profit, informal, itinerant).	Lines 125–127	Reported. We explain the reason as to why the information of the names and addresses of websites is not provided in the article.
15	Missing samples	The reasons why any outlets chosen for sampling did not furnish a sample. Do these outlets differ systematically from those in which	This research did not involve missing samples.	Not Applicable

		samples were obtained?		
16	Packaging and chemistry results	- Packaging and chemistry results and their relationship- Details of products sampled—how many, in what drug classes, countries of origin, batch numbers, manufacture and expiry dates- Results for each analysis—packaging, % AI, dissolution- Additional information could be included in supplementary material	Packaging result: lines 305–330<br>Chemistry result: lines 378-388<br>Relationship: lines 403-445	Completed and reported. We explain the reason as to why the information of countries of origin, batch numbers, manufacture and expiry dates is not provided in the article.
17	Category of poor-quality medicine	A clear statement for each medicine sample detected, whether the investigators class it as genuine, counterfeit, substandard, or degraded, with an explanation as to why and whether the medicine was registered with the government in the location(s) sampled.	Lines 296-298<br>Lines 349-358<br>Lines 373-388<br>Lines 403-445<br>Lines 458-465	Completed
18	State company and address as given on packaging	If the names of companies and addresses not given, give a reason as to why this information is not provided.	Lines 125-127	Reported. We explain the reason as to why the information of manufacturer and address is not provide in the article.
19	Sharing data with MRA	Whether the data shared with the appropriate MRA and IMPACT.	Lines 117-119	Completed
20	Dissemination	Description of any non-covert packaging features that would allow others to detect counterfeit medicines. If publication is not possible, consider disseminating via Web-based supplementary material.	This result of this research did not involve counterfeit medicines.	Not Applicable
	Discussion			

21	Key results	Summarise key results with reference to study objectives	Lines 391-477	Completed
22	Limitations	Discussion of limitations of study, especially how robust the estimates of prevalence are and how applicable they may be to wider geographical areas. Discuss the direction and extent of any potential bias	Lines 490-512	Completed
23	Interpretation	An interpretation of the results, in conjunction with prior studies, in relation to public health.	Lines 478-489	Completed
24	Intervention	Whether interventions are thought appropriate and, if so, what type	This research did not involve intervention.	Not Applicable
	Other Information			
25	Conflict of interest	State any potential conflicts of interest.	Lines 534	Completed
26	Funding	Give the source of funding and role of funders in the study.	Lines 530-532	Completed
