# Supplementary material for: Circulation of COVID-19-Related Medicines on Japanese Websites during the COVID-19 Pandemic and Their Quality and Authenticity
Source: Am J Trop Med Hyg. 2024 Sep 17;111(5):1097–106. doi: 10.4269/ajtmh.23-0710 (PMC11542516; doi:10.4269/ajtmh.23-0710)
Supplement: Supplemental Table 3 [file tpmd230710.SD3.pdf]

Supplemental Table 3. Validation parameters of dexamethasone quantified using HPLC.

**Validation parameters**		**Dexamethasone**
Linearity expressed as R^2		0.99825
Range (µg/mL)		25.0 -200.0
Precision	Intraday (RSD %)	0.1-0.3
	Inter-day (RSD%)	0.9-1.6
Recovery(%)		103.0
Accuracy	100%(6 injection)<br>[95% Confidence Interval]	[-0.0000394-0.00852]
Limit of detection[a](µg/mL)		8.2
Limit of quantitation[b](µg/mL)		24.9
Specificity		Specific

[a]The limit of detection (LOD) was determined based on a signal-to-noise ratio of 3.3:1.

[b]The limit of quantification (LOQ) was determined based on a signal-to-noise ratio of 10:1.
