# Supplementary material for: Circulation of COVID-19-Related Medicines on Japanese Websites during the COVID-19 Pandemic and Their Quality and Authenticity
Source: Am J Trop Med Hyg. 2024 Sep 17;111(5):1097–106. doi: 10.4269/ajtmh.23-0710 (PMC11542516; doi:10.4269/ajtmh.23-0710)
Supplement: Supplemental Table 4 [file tpmd230710.SD4.pdf]

Supplemental Table 4. Validation parameters of ivermectin quantified using HPLC.

Validation parameters		Ivermectin
Linearity expressed as R^2		0.99869
Range(μg/mL)		4.6-18.4
Precision	Intraday (RSD%)	0.1-1.1
	Inter-day (RSD%)	0.4-1.1
Recovery(%)		99.8
Accuracy	100% (6 injection)<br>[95% Confidence Interval]	[-0.0001215-0.000073]
Limit of detection[a](μg/mL)		0.7
Limit of quantitation[b](μg/mL)		2.2
Specificity		Specific

[a]The limit of detection (LOD) was determined based on a signal-to-noise ratio of 3.3:1.

[b]The limit of quantification (LOQ) was determined based on a signal-to-noise ratio of 10:1.
