# Supplementary material for: Circulation of COVID-19-Related Medicines on Japanese Websites during the COVID-19 Pandemic and Their Quality and Authenticity
Source: Am J Trop Med Hyg. 2024 Sep 17;111(5):1097–106. doi: 10.4269/ajtmh.23-0710 (PMC11542516; doi:10.4269/ajtmh.23-0710)
Supplement: Supplemental Table 5 [file tpmd230710.SD5.pdf]

Supplemental Table 5. Results of quantity, content uniformity, and dissolution testing of dexamethasone tablets

Japanese regular products	Quantitative analysis		Content uniformity test		Dissolution test		Any fail
Decadron® 0.5mg tablet	Mean content (%) ±SD	Judgment	Acceptance value	Judgment	Mean dissolution rate (%) ±SD	Judgment	Judgment
	103.0 ± 2.8	Pass	8.1	Pass	98.4 ± 9.8	Pass	Pass
