# Supplementary material for: Circulation of COVID-19-Related Medicines on Japanese Websites during the COVID-19 Pandemic and Their Quality and Authenticity
Source: Am J Trop Med Hyg. 2024 Sep 17;111(5):1097–106. doi: 10.4269/ajtmh.23-0710 (PMC11542516; doi:10.4269/ajtmh.23-0710)
Supplement: Supplemental Table 6 [file tpmd230710.SD6.pdf]

Supplemental Table 6. Contents (mg/tablet) and dissolution rates of stated active pharmaceutical ingredients (APIs) in the tablets.

Japanese regular products	Mean content(%)±SD[a] (n = 3[b])	Mean dissolution rate (%)±SD[a] (n = 3[b])
Stromectol® 3mg tablet	99.8 ± 1.9	96.5 ± 1.3

[a]SD, standard deviation

[b] Only 3 tablets were analyzed in this test because of the shortage of ivermectin tablet in Japan in 2022, the purchase quantity was limited.
